# Features of *Streptococcus agalactiae* strains recovered from pregnant women and newborns attending different hospitals in Ethiopia

**DOI:** 10.1186/s12879-020-05581-8

**Published:** 2020-11-16

**Authors:** Musa Mohammed Ali, Yimtubezinash Woldeamanuel, Daniel Asrat, Demissie Assegu Fenta, Bernard Beall, Stephanie Schrag, Lesley McGee

**Affiliations:** 1 Hawassa University College of Medicine and Health Sciences, School of Medical laboratory Science, Hawassa, Ethiopia; 2 Department of Microbiology, Immunology and Parasitology, Addis Ababa University College of Health Science, Addis Ababa, Ethiopia; 3 Respiratory Diseases Branch, Centers of Disease Control and Prevention (CDC), Atlanta, USA

**Keywords:** *S. agalactiae*, GBS, Sequence type, Serotype, Antibiotic resistance

## Abstract

**Background:**

*Streptococcus agalactiae* (Group B Streptococcus, GBS) serotypes, sequence types, and antimicrobial resistance profile vary across different geographic locations affecting disease patterns in newborns. These differences are important considerations for vaccine development efforts and data from large countries in Africa is limited. The aim of this study was to determine serotypes and genotypes of GBS isolates from pregnant women and their newborns in Ethiopia.

**Methods:**

A hospital based cross-sectional study was conducted at three hospitals in Ethiopia from June 2014 to September 2015. Out of 225 GBS isolates, 121 GBS were recovered, confirmed and characterized at CDC’s Streptococcus Laboratory using conventional microbiology methods and whole genome sequencing.

**Results:**

Of the 121 isolates, 87 were from rectovaginal samples of pregnant women, 32 from different body parts of their newborns and 2 from blood of newborns with suspected sepsis. There were 25 mother-infant pairs and 24 pairs had concordant strains. The most prevalent serotypes among mothers and/or their babies were II, Ia and V (41.5, 20.6, 19.5 and 40.6%, 25 and 15.6%, respectively). Multilocus sequence typing (MLST) on 83 isolates showed ST10 (24; 28.9%) and ST2 (12; 14.5%) as most predominant sequence types. All GBS strains were susceptible to penicillin, cefotaxime and vancomycin, which correlated to the presence of wildtype PBP2x types and the lack of known vancomycin-resistance genes. Tetracycline resistance was high (73; 88%, associated primarily with *tetM*, but also *tetO* and *tetL)*. Five isolates (6%) were resistant to erythromycin and clindamycin and 3 isolates were fluoroquinolone-resistant, containing associated mutations in *gyrA* and *parC* genes. All isolates were positive for one of four homologous Alpha/Rib family determinants and 1–2 of the three main pilus types.

**Conclusions:**

Predominant serotypes were II, Ia, and V. A limited number of clonal types were identified with two STs accounting for about half of the isolates. All strains collected in this study were susceptible to beta-lactam antibiotics and vancomycin. Typical of most GBS, these isolates were positive for single alpha-like family protein, serine-rich repeat gene, as well as 1–2 pilus determinants.

**Supplementary Information:**

The online version contains supplementary material available at 10.1186/s12879-020-05581-8.

## Background

Group B Streptococcus (GBS) is a recognized cause of infant sepsis and meningitis globally and is a leading cause of morbidity and mortality in Africa [1]. GBS is also a common commensal colonizer of the gastrointestinal and urogenital tracts of women and maternal colonization is a major factor in mother-to-child GBS transmission. Early onset (< 7 days) GBS disease has been well characterized, whereas the epidemiology of late onset disease (LOD, 7–89 days) is less well understood [2].

Penicillin G is the drug of choice for intrapartum prophylaxis [3] and GBS isolates remain mostly susceptible to penicillin. Its prophylactic use has been instrumental in significantly reducing the incidence of early-onset diseases in neonates [4]. However, GBS isolates with reduced susceptibility to penicillin have been reported for more than a decade [5]. Erythromycin and clindamycin have been used as alternatives to prevent vertical transmission of GBS among pregnant women who are allergic to penicillin, but resistance to these antibiotics has emerged in several countries, including reports from Africa [6, 7].

GBS strains are subdivided according to type-specific capsular polysaccharides into 10 unique serotypes which are also a major focus of vaccine development [8]. Serotypes I to V account for about 98% of colonizing GBS isolates worldwide with serotype III usually associated with invasive disease and less common among colonization isolates. GBS serotype distribution is not uniform across different geographic regions and temporal variations have also been described [9]. As information on GBS from their genomes has accumulated, molecular methods have also proved very useful for investigating the population structure of GBS and discriminating differences between strains isolated from different sources [5, 10–12]. For example, the ST17 lineage has been associated with neonatal infections, particularly with late-onset disease [11]. There are previous reports showing diversification and shifts in serotypes [5, 12, 13] and abundant evidence of past capsular switching within several MLST-based lineages [12, 14] which also pose potential challenges for the development of a vaccine. Global data on circulating GBS strains is important for disease control and for informing the development of effective vaccines. However relatively few studies are available from Sub-Saharan Africa [1] and there are limited data on serotypes and strain characterization [7].

Ethiopia is an important country with a substantial birth cohort in Africa, and while there are previous data published from Ethiopia on antibiotic susceptibility patterns for GBS, there are limited available detailed descriptive data on circulating strains [7, 15]. Here we used whole genome sequencing to investigate serotype distribution, clonal relationships, lineage distributions, virulence factor determinants, and antimicrobial susceptibility patterns of *S. agalactiae* strains recovered from pregnant mothers and their newborns attending three hospitals in Ethiopia.

## Methods

### Study population

This prospective, cross-sectional study was conducted at 3 hospitals in Ethiopia: Adama Hospital Medical College (AHMC), Hawassa University Comprehensive Specialized Hospital (HUCSH) and Tikur Anbessa Specialized Hospital (TASH) between June 2014 and September 2015. The three hospitals were selected based on their convenience. AHMC is a rural hospital located in Adama City in the Oromia regional state. It is located 100 km due east of Addis Ababa and has a total population of 220,212. HUCSH, a rural hospital, is located in Hawassa which is the capital city of Southern Nations Nationalities and Peoples Region (SNNRP) and is located 275 km south of Addis Ababa. The total population of Hawassa town is 235,000. TASH is an urban hospital located in Addis Ababa, the capital city of Ethiopia, with a large population size of 3,384,569.

### Eligibility criteria

Pregnant women who were admitted for delivery along with their newborn were included. Pregnant women with cesarean section delivery and those who were on antibiotic treatment for the last 2 weeks prior to data collection were excluded.

Newborns who were suspected of neonatal disease, those with signs and symptoms of neonatal disease (breathing problem, reduced movement, reduced suckling, seizure, slow or increased heart rate, vomiting, increased or reduced body temperature).

### Isolation of Bacteria

Recto-vaginal swabs from 840 pregnant women, samples from the nasal area, external ear, umbilical cord or throat area of 857 newborns and blood samples from newborns suspected of neonatal disease were collected. The detail are as follows: Number of pregnant women and their newborn at AHMC, Adama were 280 and 282 respectively; data collection period was from June 2014 to October 2014. Number of pregnant women and their newborn at HUCSH, Hawassa were 280 and 292 respectively; data collection period was November 2014 to March 2014. Number of pregnant women and their newborn at TASH, Addis Ababa were 280 and 283 respectively; data collection period was March 2015 to August 2015. One hundred seventy-six newborns suspected of early onset disease (defined as occurring during the first week of life) were included from TASH, Addis Ababa; data collection period was from March 2015 to August 2015.

At each study site, to isolate GBS from pregnant women and newborns, recto-vaginal swabs from mothers and samples from the nasal area, external ear, umbilical cord or throat area of newborns were placed into Lim broth (BD Diagnostics, USA) and incubated for 18–24 h at 37 °C in CO_2_ enriched atmosphere. Then sub-cultured onto sheep blood agar (BD Diagnostics, USA) and incubated in CO_2_ enriched atmosphere at 37 °C for 18–24 h. If there was no growth, blood agar plate was re-incubated and examined after 48 h.

To isolate GBS from newborns suspected of early onset disease, about 1 ml blood was inoculated into Tryptone Soy Broth (BD Diagnostics, USA). All blood cultures were incubated aerobically at 37 °C and inspected daily for 7 days for the presence of visible microbial growth by observing any of one of the following changes: turbidity, hemolysis, gas production and coagulation of broth. Blood cultures with sign of microbial growth were sub-cultured onto blood agar (BD Diagnostics, USA). The blood agar plate was incubated aerobically in CO_2_ enriched atmosphere at 37 °C for 24–48 h.

To identify GBS, hemolytic reaction on BAP (beta-hemolytic or non-hemolytic), Gram reaction, catalase test, CAMP (Christie, Atkins, and Munch-Petersen) test and Strep B Grouping Latex (Remel, USA) were used. Isolates were stored at − 70 °C in medium containing skim milk, tryptone, glucose, and glycerol [16] and transported to the Streptococcus Laboratory at the Centers for Disease Control and Prevention for confirmation and characterization. For several analyses, where more than one isolate was available for mothers and/or babies and they had the same serotype and MLST type, only a single isolate was selected so as not to duplicate results.

### DNA extraction and whole genome sequencing

At CDC, isolates were cultured on Trypticase soy agar supplemented with 5% sheep blood (BAP). A positive CAMP test and Strep. B Grouping Latex (Remel, USA) were used to confirm isolates as *S. agalactiae*.

For whole genome sequencing (WGS), GBS isolates were cultured on BAPs and incubated overnight at 37 °C. Genomic DNA was extracted manually using a modified QIAamp DNA mini kit protocol (Qiagen, Inc., Valenica, CA, USA) (https://www.cdc.gov/streplab/downloads/pcr-body-fluid-dna-extract-strep.pdf). Nucleic acid concentration was quantified by Qubit assay (Thermo Fisher Scientific Inc., Waltham, MA, USA) and samples were sheared using a Covaris M220 ultrasonicator (Covaris, Inc., Woburn, MA, USA) programmed to generate 500-bp fragments. Libraries were constructed on the SciCloneG3 (PerkinElmer Inc., Waltham, MA, USA) using an Ovation Rapid multiplex library preparation kit with 96 dual indices (NuGEN, San Carlos, CA, USA) and quantified by KAPA qPCR library quantification method (Kapa Biosystem Inc., Wilmington, MA, USA). Short read sequences were generated with MiSeq v2 500 cycle kit (Illumina Inc). Isolate identifiers, pipeline features, and assembly metrics are listed in Supplementary Table 1. For the 119 isolates that yielded high quality sequencing metrics with contig size below 500, sequences are available in the NCBI repository and accession number provided in Supplemental Table 1.

### Serotyping and surface protein detection

A multiplex PCR assay was initially used for the direct identification of the capsular serotypes (Ia to IX) of GBS [17]. In addition, serotype was confirmed using the CDC short-read WGS bioinformatics pipeline [5, 12]. The presence of hypervirulent GBS adhesion determinant (*hvgA)*, serine-rich repeat gene (*srr*), one of four alpha-family surface protein genes (rib, alpha, alp1, alp2/3) and the pilus islands (PI-1, PI-2a, and PI-2b) were also queried through the CDC pipeline (https://github.com/BenJamesMetcalf) [12].

### Multilocus sequence typing

Seven locus MLST to assign sequence type (ST) was facilitated from whole genome sequence data with the CDC pipeline using SRST2 and database at http://pubmlst.org/sagalactiae/ [5]. eBURST was used to group isolates into lineages or clonal complexes (CCs), based upon sharing at least six of 7 MLST alleles with one or more other members [18]. The relations between STs and serotypes of GBS isolates were illustrated by the minimum spanning tree (PHYLOVIZ software version 2.0; PHYLOViZ team, Lisbon, Portugal).

### Antimicrobial susceptibility testing

Antimicrobial susceptibility patterns of GBS were tested by broth microdilution. The antimicrobials tested included penicillin, cefotaxime, erythromycin, clindamycin, levofloxacin, vancomycin, daptomycin, tetracycline, and linezolid. Isolates were classified as sensitive, intermediate, or resistant according to Clinical Laboratory Standards Institute (CLSI) guidelines [19]. Strains were determined to be multidrug resistant if resistant to ≥3 different antibiotic classes. Phenotypic MICs were compared with WGS-predictions for non-β-lactam antibiotics (except daptomycin) using sequence queries and a bioinformatics pipeline (https://github.com/BenJamesMetcalf/Spn_Scripts_Reference) for detection of resistance determinants provided in a previous study [5]. The PBP2x typing scheme used serves to flag missense mutations within the *pbp2x* gene for subsequent isolate MIC testing for beta-lactam antibiotics.

## Results

### Source, serotype and MLST profiles of *S. agalactaie*

Among 225 *S. agalactiae* isolates collected at the 3 study sites, only 121 isolates were recovered and confirmed as GBS at CDC’s Streptococcus Laboratory for further characterization. Of these 121 GBS isolates, 87 were from rectovaginal samples from healthy pregnant women, 32 from different body parts of their healthy newborns (ear, throat, and nasal), and 2 from newborns with suspected early-onset disease. Twenty-eight isolates (22.8%) were collected at AHMC, Oromia Regional state (June 2014 to October 2014); 60 (48.8%) at HUCSH, Sidama Regional State (November 2014 to March 2014) and 33 (27.3%) were from TASH, Addis Ababa, the capital city of Ethiopia (March 2015 to August 2015). Of the 121 isolates, there were 25 mother-infant pairs and nine cases with > 1 isolate for mother or baby. A total of 83 non-duplicated isolates were identified for analysis of antimicrobial susceptibility and genotypes.

**Fig. 1:**
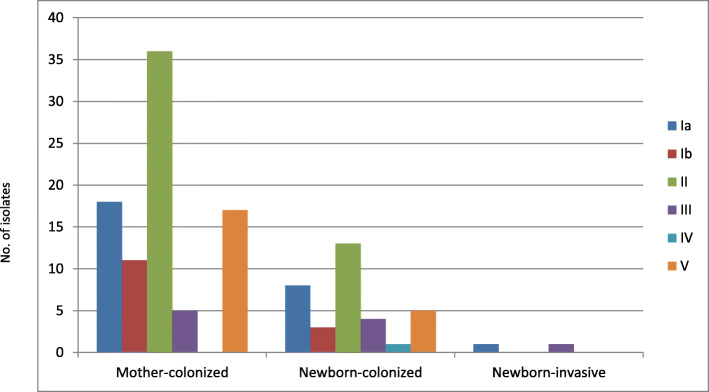
Distribution of 121 *S. agalactiae* strains collected from pregnant women (*n* = 87), their newborns (*n* = 32) and newborns suspected of early onset-disease (*n* = 2) by capsular serotype and study site (June 2014 to September 2015). Percentages within each category are shown

Six of the ten known serotypes occurred in this study population: For mother isolates: II (*n* = 36; 41.5%), Ia (*n* = 18; 20.6%), V (*n* = 17; 19.5%), Ib (*n* = 11; 12.6%), and III (*n* = 5; 5.7%) and for baby colonization isolates: II (*n* = 13; 40.6%), Ia (*n* = 8; 25%), V (*n* = 5; 15.6%), III (*n* = 4; 12.5%), Ib (*n* = 3; 9.3% and IV (*n* = 1: 3.1%) (Fig. 1). The EOD isolates from 2 newborns were Ia (ST5) and III (ST19). There was 100% concordance with serotype prediction from PCR and the genomic prediction approach.

**Fig. 2:**
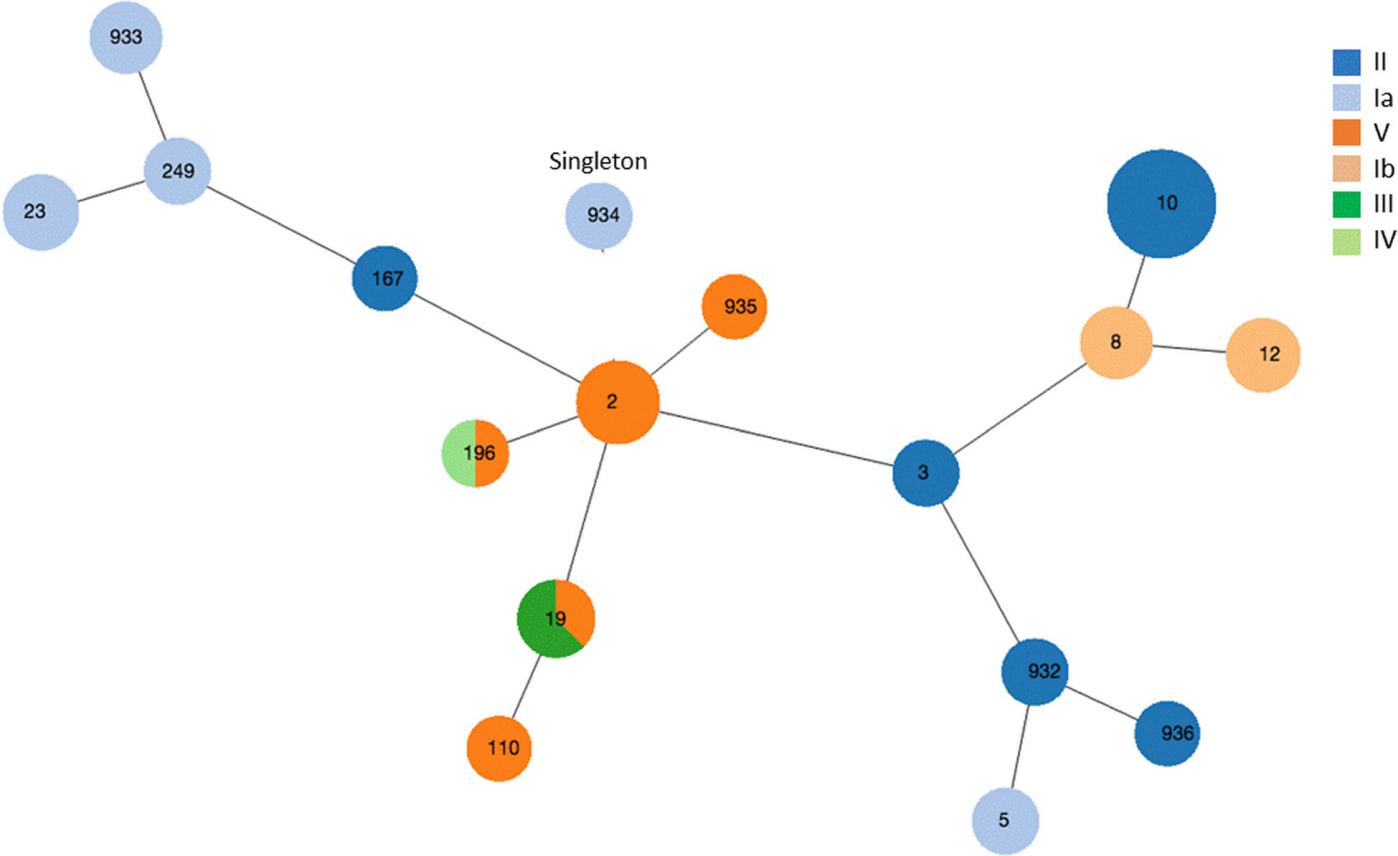
Associations of sequence types with serotypes for 83 non-duplicated *S. agalactiae* isolates. (Size of the circles are proportional to the numbers of isolates assigned to each Sequence types)

MLST analysis identified 17 different STs among 83 GBS strains. The predominant STs were ST10 (*n* = 24; 28.9%), ST2 (*n* = 12; 14.5%) and ST19 (*n* = 8; 9.6%) (Table 1). Using eBURST, the STs were grouped into 5 CCs (Fig. 2), with CC8 accounting for the majority of isolates (*n* = 36; 43.4%). One singleton (ST934) was identified and not part of a cluster. Most STs were associated with a single serotype except ST196 (represented by serotypes IV and V) and ST19 (represented by serotypes III and V). Serotype Ia was mostly associated with CC249 (*n* = 14;77.8%) and serotypes V and II with CC2 (*n* = 18; 100%) and CC10 (*n* = 24; 80%), respectively.

**Table 1:** Characteristics of the clonal complexes (CCs) and sequence types (STs) of 83 (non-duplicated isolates) *S. agalactiae* strains by serotype, resistance determinants and other strain characteristics (June 2014 to September 2015)

Clonal Complex	STs (n)	Capsular Serotype	Macrolide/lincosamide genes	Tetracycline resistance genes	***gyr***A and ***par***C mutations associated with FQ resistance	Gene coding for Alpha-like proteins ***n*** = 83 (100%)	Gene coding for SRR1 ***n*** = 83 (100%)	Gene coding for pili ***n*** = 83 (100%)	Gene coding for HVGA ***n*** = 2 (2.4%)
CC2 (24)	ST2 (12)	V (12)		*tet*M (12)		*alp*1(8);*rib*(4)	*srr*1(12)	PI-1:PI-2a(12)	
	ST196 (2)	IV (1); V (1)		*tet*M (2)		*alp*1(2)	*srr1*(2)	PI-1:PI-2a(1);PI-2b(1)	
	ST935^a^ (1)	V (1)		*tet*M (1)		*rib*(1)	*srr*1(1)	PI-1:PI-2a(1)	
	ST19 (8)	III (5); V (3)	*erm*TR(2);*erm*TR:*lnu*(2) *erm*B (1)	*tet*M (8)	*gyr*A + *par*C (3)^b^	*alp*1(3); *rib*(5)	*srr*1(8)	PI-1:PI-2a(8)	
	ST110 (1)	V (1)		*tet*O(1)		*rib*(1)	*srr*1(1)	PI-2a(1)	
CC249 (14)	ST249 (2)	Ia (2)				*alp*2/3 (2)	*srr*1(2)	PI-1 (2)	
	ST23 (7)	Ia (7)		*tet*M (7)		*alp*1(7)	*srr*1(7)	PI-2a(7)	
	ST933^a^ (5)	Ia (5)				*alp*2/3 (5)	*srr*1(5)	PI-1 (5)	
CC8 (35)	ST10 (24)	II (24)		*tet*L + *tet*M (24)		*alpha*(24)	*srr*1(24)	PI-1:PI-2a(24)	
	ST12 (6)	Ib (6)		*tet*M (4)		*alpha*(6)	*srr*1(6)	PI-1:PI-2a(6)	
	ST8 (5)	Ib (5)		*tet*M (5)		*alpha*(5)	*srr*1(5)	PI-1:PI-2a (5)	
CC932 (5)	ST936^a^ (1)	II (1)		*tet*M (1)		*rib*(1)	*srr*1(1)	PI-2b(1)	
	ST932^a^ (2)	II (2)		*tet*M (2)		*rib*(2)	*srr*1(2)	PI-2b(2)	
	ST5 (2)	Ia (2)		*tet*M (2)		*alp*1(2)	*srr*1(2)	PI-2b(2)	
ST167 (1)	ST167 (1)	II (1)		*tetM* (1)		*rib*(1)	*srr*1(1)	PI-2a(1)	
ST3 (2)	ST3 (2)	II (2)		*tet*M (2)		*alpha*(2)	*srr*1(2)	PI-2a(2)	
Singletons (2)	ST934^a^ (2)	Ia (2)		*tet*M (1)		*alp*1(2)	*srr*1(2)	PI-2b(2)	*hvga* (2)

^a^ A new ST from this study; *CC* clonal complexes; *ST* sequence types; *n* number

^b^All 5 isolates also had determinants found to be associated with gentamicin resistance (*aac6*-*aph2*)

### Surface proteins

One of the four homologous alpha protein gene family queries was detected in each isolate: *alpha* (37: 44.6%), *alp1* (24: 28.9%), *alp2/3* (7: 8.4%) or *rib* (15; 18.1%). The serine-rich repeat glycoprotein determinant *srr1* was identified in all GBS isolates and 1–2 of the three pilus backbone determinants, each of which corresponds to a distinct pilus and pathogenicity island, were also detected in all isolates: PI-1 + PI-2a (57; 68.7%), PI-2a (11; 13.3%), PI-2b (8; 9.6%), PI-1 (7: 8.4%). Two serotype Ia/ST934 isolates (2.4%) were *hvgA*-positive. (Table 1).

Serotype Ia isolates were *alp1*-positive (61.1%) or *alp2/3*-positive (38.9%). All serotype Ib and III isolates were *alpha*-positive and *rib*-positive, respectively. The majority of serotype II were *alpha*-positive (86.7%) with 13.3% *rib*-positive. Similarly, most of serotype V were *alp1*-positive (66.7%) with a smaller number having *rib* determinant (33.3%) (Table 1).

Most isolates were positive for pilus subunit queries PI-1 and PI-2a (24 serotype II, 17 serotype V, 11 serotype Ib, and 5 serotype III), while seven isolates (all serotype Ia) were positive for PI-1 alone. Isolates with serotypes Ia (*n* = 7), II (*n* = 3) and V (*n* = 1) were PI-2a positive. The pilus subunit query for PI-2b was identified in serotype Ia (4 isolates), II (3 isolates) and in a single serotype IV isolate in the study (Table 1).

### Antimicrobial susceptibility profile, resistance determinants, virulence factor determinants

All 83 non-duplicated GBS strains collected in the study were susceptible to penicillin, linezolid, cefotaxime, and vancomycin (Table 2). PBP2X types were restricted to types, 1, 4 and 5. The majority of isolates were resistant to tetracycline (*n* = 73; 88%) conferred by *tet*M (48; 57.8%), *tet*L + M (24; 28.9%) or *tet*O (1; 1.2%) (Table 1). All GBS strains of serotypes II, III, IV and V were resistant to tetracycline (Table 2). Five isolates within serotypes III and V were non-susceptible to erythromycin. These included 4 inducibly clindamycin-resistant isolates carrying *erm*TR and one constitutively clindamycin-resistant *ermB-*positive strain. Two isolates with *erm*TR were also positive for *lnu* genes. Three isolates of serotype V/ST19 had levofloxacin MICs > 4 μg/ml and had mutations within both *gyr*A and *par*C (Table 1) and these same isolates also had the *aac6* (*aph2*) determinant that confers gentamicin resistance. Five isolates were considered MDR and this included the 3 resistant to levofloxacin.

**Table 2:** Antimicrobial susceptibility patterns of 83 *S. agalactiae* strains collected from Ethiopia by serotype (June 2014 to September 2015)

Antibiotics	MIC Range (μg/ml)	N (%)	Susceptible n (%)	Intermediate n(%)	Resistant/Non-susceptible* n(%)	Serotypes, Total ***S. agalactiae*** = 83 (% based on MIC group)					
						Ia, ***n*** = 18	Ib, ***n*** = 11	II, ***n*** = 30	III, ***n*** = 5	IV, ***n*** = 1	V, ***n*** = 18
Penicillin	≤0.03	4 (4.82)	83 (100)				3 (75)	1 (25)			
	0.06	47 (56.6)				6 (12.8)	22 (46.8)	4 (8.5)		9 (19.2)	6 (12.8)
	0.12	32 (38.6)				12 (37.5)	5 (15.6)	5 (15.6)		1 (3.1)	9 (28.1)
Cefotaxime	≤0.12	81 (97.6)	83 (100)			17 (20.9)	11 (13.6)	29 (35.8)	5 (6.2)	1 (1.2)	18 (22.2)
	0.25	2 (2.4)				1 (50)		1 (50)			
Levofloxacin	≤0.5	8 (9.6)	79 (95.2)				1 (12.5)	4 (50)	2 (25)	1 (12.5)	
	1	66 (79.5)				16 (24.2)	10 (15.2)	22 (33.3)	3 (4.5)		15 (22.7)
	2	5 (6.0)				2 (40)		3 (60)			
	> 4	4 (4.8)			4 (4.8)			1 (25)			3 (75)
Tetracycline	≤1	10 (12)	10 (12)			8 (80)	2 (20)				
	> 8	73 (88)			73 (88)	10 (13.7)	9 (12.3)	30 (41.1)	5 (6.9)	1 (1.4)	18 (24.6)
Clindamycin	≤0.12	78 (98.8)	78 (94)			18 (23.1)	11 (14.1)	30 (38.5)	4 (5.1)	1 (1.3)	14 (17.9)
	32	5 (1.2)			5 (6)^a^				1 (20)		4 (80)^d^
Erythromycin	≤0.25	78 (90.2)	78 (94)			18 (23.1)	11 (14.1)	30 (38.5)	4 (5.1)	1 (1.3)	14 (17.9)
	0.5	1 (0.83)		1 (1.2)					1 (100)^c^		
	2	3 (4.1)									3 (100)
	> 32	1 (0.83)			4 (4.8)						1 (100)
Daptomycin	≤1	80 (96.4)	80 (96.4)			18 (22.5)	10 (12.5)	28 (35)	5 (6.2)	1 (1.3)	18 (22.5)
	> 1	3 (3.6)			3 (3.6)^b^		1 (33.3)	2 (66.7)			
Vancomycin	≤0.5	53 (63.9)	83 (100)			12 (22.6)	6 (11.3)	17 (32.1)	3 (5.7)	1 (1.9)	14 (26.4)
	1	30 (36.1)				6 (20)	5 (16.7)	13 (43.3)	2 (6.7)		4 (13.3)
Linezolid	≤2	83 (100)	83 (100)			18 (21.7)	11 (13.3)	30 (36.1)	5 (6)	1 (1.2)	18 (21.7)

^a^Four isolates that were clindamycin susceptible were positive for D-zone test and were considered inducibly resistant

^b^For daptomycin CLSI has only susceptible (S) interpretation MICs > 1 considered non-susceptible

^c^One serotype III isolates that were clindamycin susceptible were positive for D-zone test and were considered inducibly resistant

^d^Three serotype V isolates that were clindamycin susceptible were positive for D-zone test and were considered inducibly resistant

## Discussion

Vaccination of pregnant women against GBS is a promising strategy to prevent invasive GBS disease in their infants [8]. Vaccine candidates include protein-based formulations and serotype-specific polysaccharide-protein conjugates [20] and thus an understanding of serotype and surface-protein antigen distribution in maternal colonization and infant disease worldwide is important. While there have been several reports published from Ethiopia on maternal and infant GBS colonization and disease over the past decade, little information on GBS strain characteristics has been described. In this study, serotype II was the most common predicted serotype, with five types (Ia, Ib, II, III and V) accounting for the vast majority of isolates. This is consistent with maternal colonization serotypes described from other studies in Africa and globally [7, 9], however, serotype II is not usually the predominant serotype but rather serotypes III and V [9]. Globally, the vast majority of invasive and colonizing GBS isolates are grouped into five CCs (CC1, CC10, CC17, CC19, and CC23) [21, 22]. In this study, 88% of isolates were grouped into one of these CCs, although no isolates belonging to CC1 and CC17 were identified, emphasizing the diversity of *S. agalactiae* in human isolates and highlighting the potential for local geographic differences. A recent study from Northwest Ethiopia grouped GBS in four CCs (CC1, CC10, CC19 and CC23) [23]. Similar to findings by others [21, 22] we also saw evidence of past capsular locus replacement events within 2 genetic lineages (ST19 and ST196) which may have implications for vaccine development strategies.

Surface protein antigens play an important role in the pathogenesis of GBS infection, and several of these antigens have been documented as promising vaccine targets [24]. Our data are consistent with past work indicating that a vaccine containing the 3 pilus protein components could be effective in preventing disease caused by GBS as all strains carried at least one of the pilus proteins [25]. Similarly, in all of the isolates one of four highly related Alpha family proteins (Alpha, Rib, Alp2/3, Alp1) were detected suggesting possible broad coverage of the fused N terminal domain Rib-Alpha (GBS-NN) vaccine tested in phase I clinical trials in pregnant women [26]. The HvgA surface-anchored protein has been found to be critical for GBS intestinal colonization and translocation across the blood brain barrier during the onset of meningitis [27]. There were no isolates from meningitis cases in this study but a small proportion of GBS colonization isolates (2.4%) contained the *hvgA* gene. The *hvgA* gene has been detected in previous studies, primarily among highly virulent *S. agalactiae* belonging to serotype III/ST17 [27] and rarely found among non-serotype III isolates [14, 28]. Two *hvgA* positive isolates in our sampling were of serotype Ia/ST934, which is genetically divergent from other characterized GBS. The closest MLST match within the global database was the triple locus variant ST1232 from an invasive, *hvgA-*negative serotype II strain recovered during 2017 [12http://pubmlst.net].

*S. agalactiae* have generally been considered universally susceptible to penicillin although there have been several reports of isolates with mutations associated with decreased susceptibility over the last decade [12, 29, 30]. Here, PBP 2X types were restricted to types, 1, 4 and 5, commonly seen in penicillin-susceptible US isolates [5] and all isolates were sensitive to penicillin and cefotaxime. This supports data published from Jimma, Ethiopia [31], however, several previous colonization studies in Ethiopia have documented large numbers of GBS strains resistant to penicillin [15, 32]. Differences in these reported rates warrants further investigation to determine if these rates are indeed real or due to challenges with appropriate and accurate laboratory testing for GBS species identification and antibiotic resistance. The proportion of isolates with in vitro resistance to both erythromycin and clindamycin was similar to rates described by Mengist et al. [31] but lower than that reported from other regions of Ethiopia [7, 32] and other countries [7, 12, 33]. Combined resistance to erythromycin and clindamycin in GBS is most commonly due to 23S rRNA methylases encoded by different *erm* genes which supports our findings with *erm*TR and *erm*B determinants predominantly associated with macrolide and lincosamide resistance [5]. The high level of tetracycline resistance, associated with *tet*M resistance determinant, may strengthen the hypothesis that current globally circulating *S. agalactiae* strains in humans were selected by tetracycline usage in 1940s [34]. The proportion of isolates resistant to levofloxacin were similar to that from United States [5, 12], Taiwan [35], Italy [36] and Brazil [37] and lower compared to data reported from China [38] and Canada [39].

A key strength of our study was the molecular characterization of GBS isolates from a region of the world with limited data on this subject. A major challenge and limitation were the reduced recovery rate of GBS strains at CDC allowing only half of the isolates available for additional testing. The inability to recover GBS from the initial frozen stocks could partly be due to loss of viability during storage and transport, and a number of samples were also heavily contaminated which may have contributed also to reduced recovery.

## Conclusions

In summary, the most prevalent GBS serotype was serotype II followed Ia, V, Ib, III and IV. This study suggests that circulating GBS in mothers and infants is primarily restricted to five major genetic lineages. All isolates were susceptible to penicillin and resistance to macrolides was relatively low. This study highlights the importance of additional studies to assess GBS epidemiology and develop accurate GBS prevention strategies in Ethiopia. Further epidemiological studies are needed for a more detailed understanding of GBS strain distributions and for subsequent development of vaccination strategies.

## Supplementary Information

**Additional file 1:** **Supplemental Table S1.** WGS NCBI accession numbers and bioinformatics pipeline details

## Data Availability

WGS accessions numbers for 83 *S. agalactiae* isolates are as follows: SAMN14907220, SAMN14907221, SAMN14907223, SAMN14907224, SAMN14907225, SAMN14907226, SAMN14907227, SAMN14907228, SAMN14907229, SAMN14907230, SAMN14907231, SAMN14907232, SAMN14907234, SAMN14907235, SAMN14907236, SAMN14907237, SAMN14907238, SAMN14907239, SAMN14907240, SAMN14907242, SAMN14907244, SAMN14907245, SAMN14907246, SAMN14907247, SAMN14907250, SAMN14907251, SAMN14907252, SAMN14907253, SAMN14907255, SAMN14907258, SAMN14907259, SAMN14907260, SAMN14907261, SAMN14907264, SAMN14907266, SAMN14907267, SAMN14907269, SAMN14907270, SAMN14907271, SAMN14907272, SAMN14907273, SAMN14907276, SAMN14907278, SAMN14907279, SAMN14907280, SAMN14907282, SAMN14907283, SAMN14907284, SAMN14907288, SAMN14907289, SAMN14907290, SAMN14907291, SAMN14907292, SAMN14907293, SAMN14907294, SAMN14907295, SAMN14907296, SAMN14907297, SAMN14907299, SAMN14907301, SAMN14907302, SAMN14907303, SAMN14907307, SAMN14907308, SAMN14907310, SAMN14907312, SAMN14907315, SAMN14907316, SAMN14907317, SAMN14907318, SAMN14907320, SAMN14907324, SAMN14907325, SAMN14907326, SAMN14907327, SAMN14907328, SAMN14907329, SAMN14907330, SAMN14907331, SAMN14907332, SAMN14907333, SAMN14907335, SAMN14907336 (Supplemental File 1). Sequences are deposited at NCBI. The datasets used and/or analysed during the current study are available from the corresponding author on reasonable request.

## Abbreviations

GBS: Group B Streptococcus
CDC: Centers for Disease Control and Prevention
MLST: Multilocus sequence typing
ST: Sequence type
AHMC: Adama Hospital Medical College
HUCSH: Hawassa University Comprehensive Specialized Hospital
TASH: Tikur Anbessa Specialized Hospital
CAMP: Christie, Atkins, and Munch-Petersen
WGS: Whole genome sequencing
CC: Clonal complex
PBP: Penicillin binding protein
PCR: Polymerase Chain Reaction
HVGA: Hyper Virulent GBS Adhesin
